# Acceptability and practicability of self-management for patients with Parkinson’s disease based on smartphone applications in China

**DOI:** 10.1186/s12911-020-01187-x

**Published:** 2020-08-11

**Authors:** J. Hu, D. Z. Yuan, Q. Y. Zhao, X. F. Wang, X. T. Zhang, Q. H. Jiang, H. R. Luo, J. Li, J. H. Ran, J. F. Li

**Affiliations:** 1 Department of Anatomy, and Laboratory of Neuroscience and Tissue Engineering, Basic Medical College, Chongqing Medical University, No.1, Yixueyuan Road, Chongqing, 400010 China; 2 Department of Neurology, the Second Affiliated Hospital of Chongqing Medical University, No.76, Linjiang Road, Chongqing, 400010 China

**Keywords:** Parkinson’s disease, Smartphone apps, Chronic management, mHealth

## Abstract

**Background:**

China has had about 1.2 billion mobile-phone users, and this number continues to grow. However, mobile-health services (mHealth) are currently in the initial stage, and have not yet prevailed in China. Additionally, the prevalence of Parkinson’s disease (PD) in China is 1700/100,000 (≥65 years). Indeed, these PD patients would benefit from mHealth to manage their disease. Therefore, we designed a study to determine attitudes toward smartphone applications (apps) for chronic condition self-management, and to discover the practicality of these apps among PD patients in China.

**Methods:**

We selected 204 participants with PD between 52 and 87 years old and surveyed their attitudes concerning the use of smartphone apps for chronic condition management via questionnaires.

**Results:**

Among the participants, 65.19% had smartphones. Among these smartphone users, 82.84% expressed a preference for using apps for PD management. This group tended to be younger and more frequent web users with higher education and better medication compliance, and they tended to have a longer PD course and worse conditions (*P* < 0.001, *P =* 0.001, *P* < 0.001, *P* = 0.041, *P* < 0.001, *P* = 0.013). Additionally, the willingness to apply apps for PD self-management was positively related to education (*P* < 0.001) and negatively related to age and PD course (*P* = 0.017, *P* < 0.001).

**Conclusion:**

In China, patients with PD have a generally positive attitude towards self-management through smartphone apps. Consequently, improving the coverage of smartphones with practical and handy apps is a promising strategy for PD self-management.

## Background

Parkinson’s disease (PD) is a progressive neurological disorder associated with the degeneration of dopamine-producing cells in the nigra [1]. Up to now, the average prevalence and incidence of Parkinson’s disease (PD) in China have been 1700/100,000 (≥65 years) and 797/100,000 (per year) respectively, also, the number of PD prevalence is expected to reach about 5 million by 2030 [2–4]. The cardinal features of PD are bradykinesia, tremors, rigidity, and postural instability [5]. Patients suffering from PD are frequently disturbed by the motor symptoms of PD in daily functions, activities, and roles [6]. Although the pathological mechanism and treatment strategy of PD have progressed significantly in recent years, there is still no effective treatment strategy. Recent studies have indicated that smartphone applications (apps) have the potential to offer automated and customized support for medication compliance to individuals with chronic diseases [7]. Recently, a variety of apps have been developed and successfully applied for effective self-management by patients with hypertension, diabetes, and other chronic diseases [8–12]. Moreover, apps for PD self-management have been used in countries such as the Netherlands and Sweden [13, 14]. Indeed, self-management is considered one of the most effective measures to help improve PD patient medication compliance, control their clinical symptoms, and mitigate the adverse effects of the disease [13, 15].

China has about 1.2 billion cell-phone users, and this number continues to grow [16]. However, mobile health services (mHealth) are currently in their initial stages. The key to the success of any app is to cater to the willingness of the targeted individuals and to secure their acceptance, while facilitating their use of the technology [17, 18]. Thus, the purpose of our research was to investigate the acceptability and practicability of using apps for PD self-management in China.

## Methods

### Subjects and interviews

All PD patients were recruited from the Parkinson’s Clinic at the Second Affiliated Hospital of Chongqing Medical University. The patients were examined by at least two experienced neurologists. In our study, PD was determined according to the diagnosis of PD and exclusionary criteria [19]. All participants were able to read, write, and understand what they were asked, ensuring their ability to complete the questionnaire. The investigators were trained rigorously in the methodology of the study before interviewing the target patients. This study was approved by the Research Ethics Committee of the Second Affiliated Hospital. All patients signed an informed consent form when enrolling in the study.

### Questionnaire content

The questionnaire (Supplementary file) consisted of Part I, Part II, Part III, and Part IV. Part I pertained to patient characteristics, including demographic data (age, gender, educational level, occupation) and the main clinical features of PD (PD disease course, number of anti-PD drugs, Movement Disorder Society Unified Parkinson’s Disease Rating Scale (MDS-UPDRS) [20], and Hoehn & Yahr Stage (H & Y) [21]).

Part II surveyed the number of patients with mobile phones and the ways they used them to obtain general information about PD. We also investigated the preferences of patients with PD with regards to smartphone apps. According to this survey, PD patients used smartphones to seek general information about PD (symptoms, pathophysiology, epidemiology, and prognosis), to interact with doctors and ask them questions, for advice about medication types, new medicines, side effects, and symptoms, for videos and written information on the motor and non-motor symptoms of PD, for suitable rehabilitation exercises, and for adverse factors to be avoided, such as missing medication, accidental falls, emotional disorders, and sleep deprivation.

Part III evaluated medication adherence. A modified Morisky Medication Adherence Scale (MMAS-8) was used. MMAS-8 offers good test–retest reliability (intraclass correlation coefficient = 0.729) and moderate internal consistency (Cronbach’s α = 0.556) [22–24]. Seven of the eight items (Items 1–7) were yes/no questions, where “no” was given one point, and “yes” received zero points. Item 8 was measured on a five-point Likert scale, in which “never,” “once in a while,” “sometimes,” “usually,” and “all the time” were respectively scored at 1, 0.75, 0.50, 0.25, and 0 [22–25].

Part IV was a questionnaire with ten items designed to assess attitudes toward apps for self-managing PD. These items were all answered using a five-point Likert type scale: strongly agree (5), agree (4), neutral (3), disagree (2), and strongly disagree (1). The questionnaire was an improved version of questionnaires used in the studies on the use of apps for chronic disease management in patients with epilepsy and asthma [26, 27].

### Statistical analysis

SPSS 25.0 and Graphpad Prism 7.0 software was used for statistical analysis. The demographics and clinical characteristics of the patients were analyzed according to the mean ± standard deviation (SD) for continuous variables and by frequency distributions for categorical variables. Attitudes toward smartphones were analyzed using the Student’s *t*-test for continuous variables, Pearson chi-squared test for categorical variables, and Spearman correlation analysis.

## Results

### Demographics and clinical characteristics

Between January 2017 and May 2018, a total of 208 patients were asked to participate in the survey. Of these, 204 participants (115 male and 89 female) agreed. The average age of the participants was 68.75 ± 9.54 years, ranging from 52 to 87 years old. The number of patients who lived in cities and rural areas was 172 (84.31%) and 32 (15.69%), respectively. The mean education level of the participants was 5.20 ± 3.14 years (from 1 year to 15 years). In addition, 71.08% of patients took no more than two types of anti-PD drugs. The mean Morisky Scale score was 5.75 ± 1.45. The demographic details of the patients are given in Table 1.

**Table 1:** Demographic and Parkinson’s disease-related clinical characteristics of surveyed participants

Variable	No.	%
Age (years)		
≤ 60	46	22.55
61–70	84	41.76
≥ 71	74	36.27
Gender		
Male	115	56.37
Female	89	43.63
Resident location		
Urban	172	84.31
Rural	32	15.69
Education level		
≤ 6	39	19.12
7–12	127	62.25
≥ 13	38	18.63
Occupation (employment)		
self-employed	32	15.69
Stable work or retirees	172	84.31
Number of anti-PD drug		
≤ two drugs	145	71.08
≥ three drugs	59	28.92
PD course		
≤ 5	133	65.20
≥ 6	71	34.80
MDS-UPDRS (Hoehn & Yahr Grade)		
≤ 50(I ~ II)	115	56.37
51–100(III)	89	43.63

### Mobile phone usage and means of obtaining PD information

**Fig. 1:**
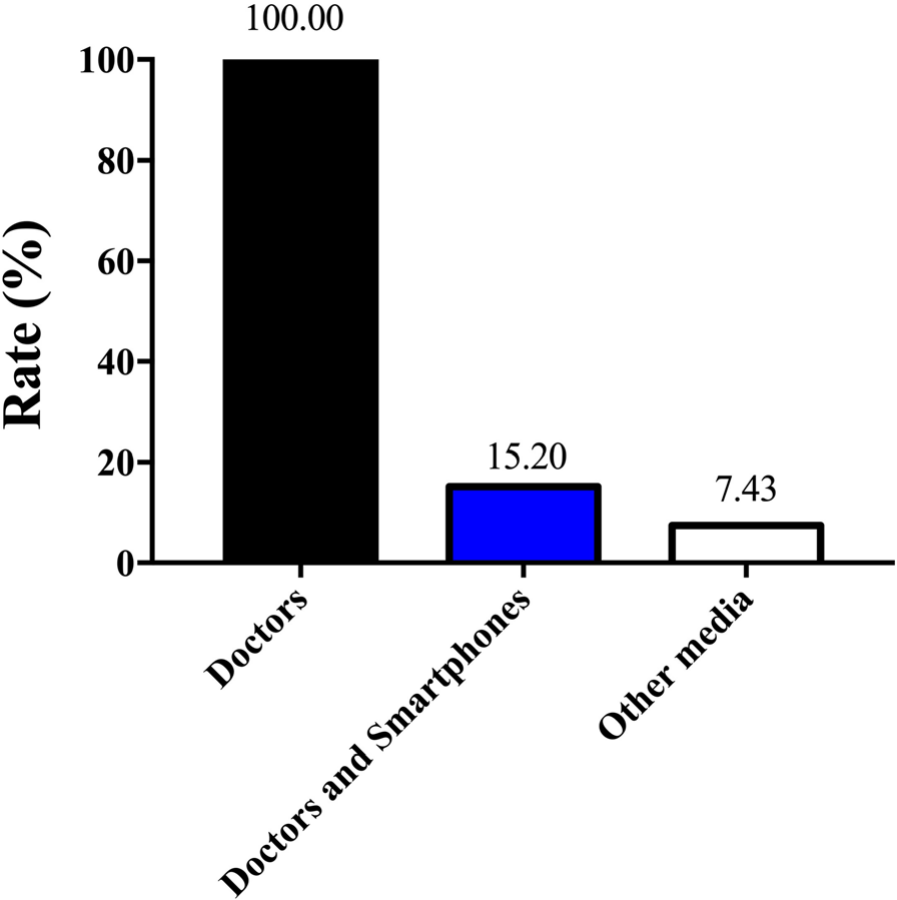
Methods of obtaining Parkinson’s disease information

Almost all respondents (96.08%, 196/204) owned cell phones, and most patients (65.19%, 133/204) owned smartphones or had access to them. According to our survey, 50.00% (102/204) of patients browsed the web; 20.59% (21/102) of these used computers to do so, while 79.41% (81/102) used smartphones. Respondents claimed they obtained PD information from doctors in clinics (100%, 204/204), from smartphones (15.20%, 31/204), and through other media (7.43%, 16/204) (Fig. 1).

### Willingness and attitude toward PD self-management apps

Only 8.82% (18/204) of participants had heard of apps for managing chronic diseases such as diabetes mellitus or hypertension. However, more than half of the surveyed patients indicated that they would use apps for PD self-management given the following: the apps are provided for free and are useful and easy to operate; the apps can be used to remind the user to take medication on time; the user’s privacy is protected; and the apps reduce economic and psychological burden (Table 2). Most importantly, the participants had a positive attitude toward using PD self-management apps, provided that the apps are easy to operate.

**Table 2:** Survey results of reaction to the Parkinson disease management apps for related patients

Survey items	SA + A N (%)	N N (%)	SDA + DA N (%)
I would use it, if it were free.	136	47	21
	66.67	23.04	10.29
I would try it out, if it were easy to operate.	169	20	15
	82.84	9.81	7.35
I would use it, if it allowed doctor to make medication change quicker.	145	41	18
	71.09	20.09	8.82
I would use it, if it protected my privacy.	139	22	43
	68.14	10.78	21.08
I think it will solve the questions related to Parkinson’s disease.	124	66	14
	60.78	32.35	6.86
I think it will help remind me to follow doctors’ directions.	150	32	22
	73.53	15.69	10.78
I think it will reduce the psychological burden of Parkinson’s disease.	160	26	23
	75.98	12.75	11.27
I think it will reduce the frequency of seeking medical advice and the costs.	148	37	19
	72.55	18.14	9.31
I believe it well be helpful for me to communicate with doctor.	151	18	37
	73.04	8.82	18.14
I think it will be useful to manage my Parkinson’s disease.	141	63	3
	67.65	30.88	1.47

*SA* strongly agree, *A* agree, *N* neutral, *SDA* strongly disagree, *D* disagree, *SD* standard deviation

### Participant interest in smartphone app content for PD management

**Fig. 2:**
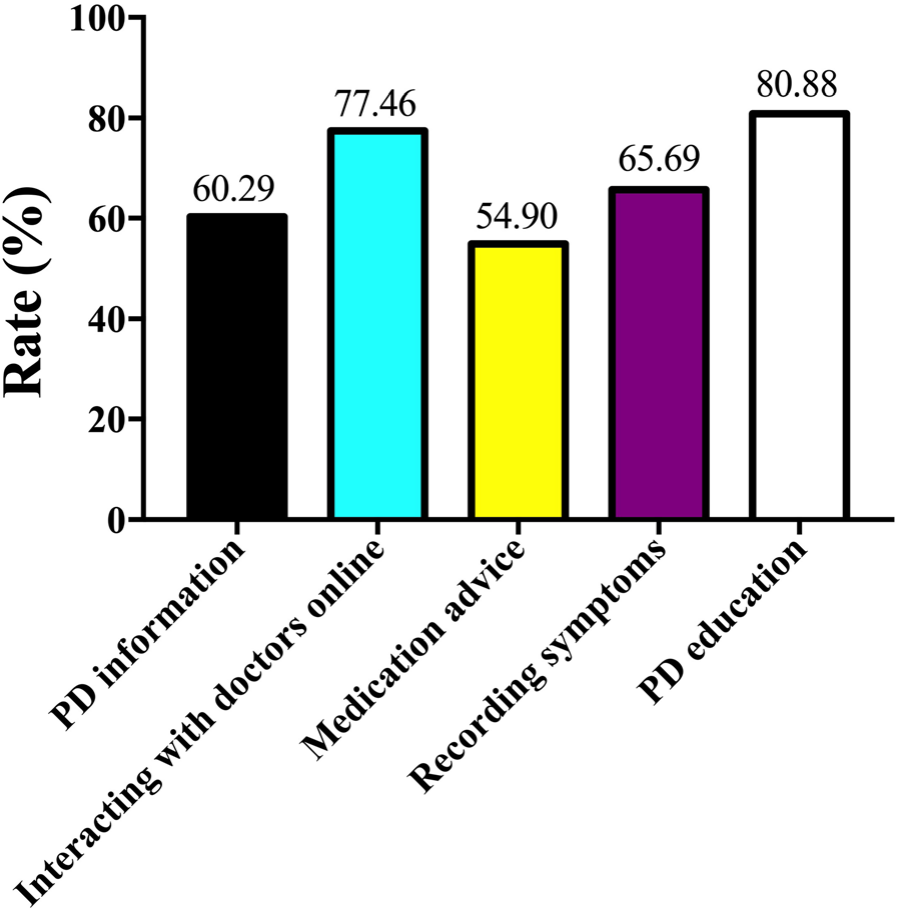
Participant interest in content of smartphone apps for PD self-management

The interest of the participants in various content on smartphone apps for PD self-management is depicted in Fig. 2. The percentage of patients interested in general PD information, interacting with doctors online, medication advice, recording symptoms, and PD education was 60.29, 77.46, 54.90, 65.69, and 80.88%, respectively.

### Characteristics of respondents with positive attitudes to using apps

To better understand the characteristics of potential users, we analyzed the correlation between each item related to patient attitudes toward smartphone apps. In particular, we measured the survey item, “I would try it out, if it were easy to operate.” This item reflects a key point concerning patient benefits. The demographic and clinical characteristics of the participants are shown in Table 3. Among these patients, those who tended to use apps were younger and better educated, they had good medication adherence, they tended to browse the web, and they had a longer PD course with worse PD conditions (*P* < 0.001, *P* = 0.001, *P* < 0.001, *P* = 0.041, *P* < 0.001, *P* = 0.01). In regard to willingness to use apps, there were no statistically differences between women and men (*P* = 0.517), resident location (urban or rural, *P* = 0.795), occupation (self-employed or stable work/retired, *P* = 0.478), or drug intake number (no more than two or no fewer than three anti-PD drugs, *P* = 0.162).

**Table 3:** Demographic and clinical characteristics of survey patients who would like to use a Parkinson’s disease self-management app, provided it is easy to operate

Variable	SA + A	N + SD + D	*P*
Age (years)	67.05 ± 9.24	76.14 ± 9.76	< 0.001
Education level	10.81 ± 3.23	8.83 ± 3.26	0.001
Morisky Scale score	5.91 ± 1.41	4.94 ± 1.39	< 0.001
Gender			0.517
Male/Female	97/72	18/17	
Resident location			0.795
*Urban*	143 (84.6%)	29 (82.9%)	
*Rural*	26 (15.4%)	6 (17.1%)	
Occupation (employment)			0.478
*self-employed*	57 (33.7%)	14 (40.0%)	
*Retirees*	112 (66.3%)	21 (60.0%)	
Number of anti-Parkinson drug			0.162
*≤ two drugs*	123 (72.8%)	22 (61.1%)	
*≥ three drugs*	46 (27.2%)	14 (38.9%)	
Whether browsing a web			0.041
*Browsing*	90 (53.3%)	12 (34.3%)	
*Not browsing*	79 (46.7%)	23 (65.7%)	
Parkinson disease course			< 0.001
*≤ 5 years*	121 (71.6%)	12 (36.4%)	
*≥ 6 years*	48 (28.4%)	21 (63.6%)	
MDS-UPDRS (H & Y)			0.013
*≤ 50(I-II)*	87 (51.5%)	26 (74.3%)	
*51–100(III)*	82 (48.5%)	9 (25.7%)	

To further investigate the correlation between the characteristics of the participants and PD app acceptance, we performed a correlation analysis. The results suggest that the willingness of patients to use apps for PD management is positively related to education level (*P* < 0.001). However, the age and PD course were negatively correlated with it (*P* = 0.017, *P* < 0.001), and MDS-UPDRS was uncorrelated (*P* = 0.924). The results are shown in Table 4.

**Table 4:** Correlation analysis between demographic and clinical characteristics of survey patients and APP acceptance

	correlation coefficient	*P*
Age	−2.56	< 0.001
Education level	0.167	0.017
MDS-UPDRS	0.007	0.924
Parkinson disease course	−2.76	< 0.001

## Discussion

To our knowledge, there is no study on the practicability and acceptability of smartphone apps for PD self-management among the elderly. This study investigated the willingness of PD patients (primarily elderly) from various backgrounds to use self-management apps. Although smartphone usage among the elderly is relatively low, they expressed a clear willingness to manage PD through apps. Therefore, using apps for PD self-management is desirable.

In our study, 96.08% of the participants (196/204) owned mobile phones, and 67.35% (133/196) owned smartphones [28]. Although many patients use smartphones, few use them to search for information about PD with their phones. Furthermore, mHealth and electronic health (eHealth) apps for self-managing chronic diseases are increasingly common [9, 29]. However, most Chinese patients still acquire information about PD by asking doctors in clinics. Few patients with PD were aware of smartphone apps for self-management before being surveyed [30, 31]. In our study, moreover, relatively few PD patients used smartphones compared to the participants with other diseases in a previous study on smartphone use [18]. This may be because smartphones are relatively poorly perceived and accepted by the elderly—a demographic that is particularly vulnerable to PD. Therefore, in terms of Chinese PD patients, the popularity of smartphones needs to be further boosted.

With regards to the content on smartphone apps for PD self-management, patients are eager to gain access to PD education, record their symptoms, and communicate with doctors online through such apps. This reveals a paucity of general PD information, related medical education, and communication with doctors in China. Furthermore, patients indicated the desire to record their motor and non-motor symptoms to better self-manage PD.

The study also found that those patients who were more willing to use self-management PD apps were younger and better educated; they had higher web usage and better drug compliance; and they tended to have more severe PD symptoms for a longer duration. Indeed, younger patients with a higher level of education were more likely to appreciate the opportunities of smart devices. Similarly, patients with experience on the web tended to have a more positive attitude toward app-based self-management. Furthermore, our study found that patients with better drug compliance were more willing to use apps. This is inconsistent with the findings of Browning et al. [26], who suggested that the drug compliance of kidney-transplant patients was not related to their willingness to use apps for self-management. This inconsistency may be the result of the different social context in which the patients were located. The patients in our study were residents of China, and a different social context can result in considerable economic, cultural, and educational differences that affect patient drug compliance. In addition, it is worth mentioning that the drug compliance of the PD patients in this study was generally poor, perhaps because PD patients are older. Moreover, in this study, patients suffering from PD over a longer duration and with more severity indicated a relatively high preference for apps. This is consistent with the results of a previous study involving patients with epilepsy [27], and may be related to the need for a professional platform to obtain advice pertaining to management and control. Generally, the willingness of patients to use apps for PD management is related to age, education level, frequency of web browsing, drug compliance, disease duration, and disease severity. Therefore, app designs should consider these features in target users to improve the practicability and convenience of such apps.

The study has several limitations. First, all patients with PD were recruited from a single location. In future studies, a larger, more diverse sample should be collected. Second, we investigated patient attitudes toward smartphone apps for PD self-management, without collecting any feedback on the actual use of such apps in practice. In future research, we will assess the effectiveness of apps used to manage PD.

## Conclusion

The results of our study showed that many PD patients owned smartphones, and that there was a positive attitude toward PD-related smartphone apps. Consequently, increasing smartphone usage and developing informative and helpful apps is a promising strategy for PD self-management.

## Supplementary information

**Additional file 1 Questionnaire**.

## Data Availability

The datasets used and analyzed in the study are available from the corresponding author upon reasonable request.

## Abbreviations

mHealth: mobile health
eHealth: electronic health
SD: Standard deviation
app: smartphone application
PD: Parkinson’s disease
H & Y: Hoehn & Yahr Stage
MMAS: Morisky Medication Adherence Scale
MDS-UPDRS: Movement Disorder Society Unified Parkinson’s Disease Rating Scale
