# Supplementary material for: Acceptability and practicability of self-management for patients with Parkinson’s disease based on smartphone applications in China
Source: BMC Med Inform Decis Mak. 2020 Aug 11;20:183. doi: 10.1186/s12911-020-01187-x (PMC7418435; doi:10.1186/s12911-020-01187-x)
Supplement: Supplementary file 1 — Additional file 1 Questionnaire. [file 12911_2020_1187_MOESM1_ESM.docx]

**Supplementary file**

**Questionnaire**

**Part I:**

**Demographic and Parkinson's disease-related clinical characteristics of surveyed participants（The second half is filled by the doctor）.**

1. Name：_____________

2. Gender: □Male □Female

3. Age：_________ years

1. Telephone Number: _____________
2. Address：District_________ County/City_________ Province____________
3. Occupation：_________________
4. ID Number：_________________ 8. Marital Status: □ Unmarried □ Married
5. Education Level：□Primary school(≤6 years) □Middle school or High school（7～12years） □University or others(≥13 years)
6. Resident Location: □Rural □Urban（Including county）
7. PD course：___________________ years
8. Number of anti-PD drug: □≤two drugs □≥three drugs

**Part II:**

**Mobile phone usage and means of obtaining PD information**

1. Do you have a cell phone? □Yes □No（The answer is yes, then answer the second question）

2. You have □Non-smartphone □Smartphone

3. Do you usually surf the Internet? □Yes □No （The answer is yes, then answer question 4）

4.The most commonly usage □Cell phone □Computer （Including desktops, laptops, and tablets）

5. Access to Parkinson's disease information □Doctor (Outpatient) □Computer □Cell phone □Others（TV，Newspaper）

6. Ever heard of a mobile self-management program for chronic diseases? ( Such as managing diabetes, high blood pressure ） □Yes □No

7. I would use it, if it were free: □SA □A □N □SDA □DA

8. I would try it out, if it were easy to operate: □SA □A □N □SDA □DA

9. I would use it, if it allowed doctor to make medication change quicker:

□SA □A □N □SDA □DA

10. I would use it, if it protected my privacy: □SA □A □N □SDA □DA

11. I think it will solve the questions related to Parkinson's disease:

□SA □A □N □SDA □DA

12. I think it will help remind me to follow doctors' directions:

□SA □A □N □SDA □DA

13. I think it will reduce the psychological burden of Parkinson's disease:

□SA □A □N □SDA □DA

14. I think it will reduce the frequency of seeking medical advice and the costs:

□SA □A □N □SDA □DA

15. I believe it well be helpful for me to communicate with doctor:

□SA □A □N □SDA □DA

16. I think it will be useful to manage my Parkinson's disease:

□SA □A □N □SDA □DA

*(SA: Strongly agree; A: Agree; N: Neutral; SDA: Strongly disagree; DA: Disagree)*

Part III

According to reference [23] Reboldi G, Moon SJ, Lee W-Y, Hwang JS, Hong YP, Morisky DE: Accuracy of a screening tool for medication adherence: A systematic review and meta-analysis of the Morisky Medication Adherence Scale-8. 2766 2017, 12(11).

<https://journals.plos.org/plosone/article?id=10.1371/journal.pone.0187139>

Part IV

According to reference [25] Liu X, Wang R, Zhou D, Hong Z: Feasibility and acceptability of smartphone applications for seizure self-management in China: Questionnaire study among people with epilepsy. 2600 2016, 55:57-61.

<https://www.epilepsybehavior.com/article/S1525-5050(15)00649-6/fulltext>
